# P-1691. Toward a Standalone Diagnostic for CDI: Clinical Evaluation of an Ultrasensitive Single-Molecule Counting C. difficile Toxin A/B Assay Against CCNA

**DOI:** 10.1093/ofid/ofaf695.1865

**Published:** 2026-01-11

**Authors:** Mariya Soban, Niamh Nolan, Justin Nguyen, Veronica Luzzi, Renee Tobias, Frank Zaugg, Peter Wagner, Valerie Brachet, Johanna Sandlund

**Affiliations:** Fluxus, Inc., Sunnyvale, California; Fluxus, Inc., Sunnyvale, California; Fluxus, Inc., Sunnyvale, California; TriCore Research Institute, Albuquerque, New Mexico; Fluxus, Inc., Sunnyvale, California; Fluxus, Inc., Sunnyvale, California; Fluxus, Inc., Sunnyvale, California; Fluxus, Inc., Sunnyvale, California; Fluxus, Inc., Sunnyvale, California

## Abstract

**Background:**

*Clostridioides difficile* infection (CDI) is a toxin-mediated disease with substantial morbidity, mortality, and healthcare costs. Current diagnostic approaches lack either sensitivity (toxin EIAs) or specificity (NAATs), necessitating complex multistep algorithms. Ultrasensitive detection of toxins A and B (TcdA/B), which correlates with disease presence, may enable accurate, standalone diagnosis.

Fluxus’ optofluidic single-molecule counting platform enables pg/mL-level detection in a low-complexity format. Here, we report preliminary analytical and clinical performance of the Fluxus *C. difficile* TcdA/B assay, including testing of patient samples.

**Methods:**

Limits of detection (LoD), quantification (LoQ), dynamic range, spike recovery, and cross-reactivity of the TcdA, TcdB, and combined assays were assessed. Clinical performance was evaluated using 30 stool samples from patients with suspected CDI, previously tested with GDH/toxin EIA and NAAT (*tcdB*). Reference testing was performed using cell cytotoxicity neutralization assay (CCNA). The clinical threshold for positivity for the Fluxus *C. difficile* TcdA/B assay was established using Youden Index optimization. Positive percent agreement (PPA) and negative percent agreement (NPA) with CCNA were calculated based on this threshold using the same dataset.

**Results:**

LoDs for TcdA, TcdB, and combined assays were 0.015, 1.47, and 0.35 pg/mL, respectively; LoQs were 0.33, 5.08, and 1.66 pg/mL. Mean spike recovery for TcdB was 102% (range 67–120%), and no crossreactivity between TcdA and TcdB was observed. Of 30 clinical samples with suspected CDI, 11 were CCNA positive and 19 were CCNA negative. Using the optimized clinical threshold, the ultrasensitive Fluxus assay demonstrated 100% PPA and 100% NPA with CCNA. In contrast, toxin EIA misclassified one CCNA-positive sample as negative, while NAAT identified six CCNA-negative samples as positive.

**Conclusion:**

The ultrasensitive Fluxus *C. difficile* toxin A/B assay demonstrated perfect agreement with CCNA in this sample set, outperforming both GDH/toxin EIA and NAAT. While these findings support the assay’s potential as a standalone diagnostic for CDI with high sensitivity and specificity, confirmation in an independent sample set is needed to validate performance.

**Disclosures:**

Mariya Soban, MS, Fluxus: Employee Niamh Nolan, MS, Fluxus: Advisor/Consultant|Fluxus: Advisor/Consultant Justin Nguyen, BS, Fluxus: Employee|Fluxus: Employee Renee Tobias, MS, Fluxus: Employee Frank Zaugg, PhD, Fluxus: Employee Peter Wagner, PhD, Fluxus: Employee Valerie Brachet, PhD, Fluxus: Employee Johanna Sandlund, MD, PhD, Fluxus: Employee

